# Catheter-directed thrombolytic therapy combined with angioplasty for hepatic vein obstruction in Budd-Chiari syndrome complicated by thrombosis

**DOI:** 10.3892/etm.2013.1239

**Published:** 2013-07-30

**Authors:** QINGQIAO ZHANG, HAO XU, MAOHENG ZU, YUMING GU, NING WEI, WENLIANG WANG, ZHIKANG GAO, BIN SHEN

**Affiliations:** Department of Interventional Radiology, The Affiliated Hospital of Xuzhou Medical College, Xuzhou, Jiangsu 221006, P.R. China

**Keywords:** Budd-Chiari syndrome, hepatic vein thrombosis, thrombolytic therapy, hepatic vein angioplasty/stenting

## Abstract

The aim of this study was to assess the efficacy and safety of catheter-directed thrombolysis combined with angioplasty in the treatment of hepatic vein obstruction in Budd-Chiari syndrome (BCS) complicated by thrombosis. In 14 cases of BCS, the patients with hepatic vein obstruction complicated by thrombosis who underwent catheter-directed urokinase thrombolysis, balloon dilatation and/or stent placement were followed up with an ultrasound examination of the liver. Among the 13 cases of successful treatment, one hepatic vein was recanalized in 12 patients (right hepatic vein, seven cases; left hepatic vein, three cases; middle hepatic vein, one case and accessory hepatic vein, one case) and two hepatic veins (right and left) were recanalized in one patient without serious complications, such as bleeding and pulmonary embolism. There was one patient in whom the treatment was unsuccessful. During an average follow-up period of 24.8±19.6 months, hepatic vein restenosis was observed in one patient in the sixth month after opperation; however, a successful result was obtained following a second balloon dilatation. The remaining 12 patients did not demonstrate any recurrence of restenosis or thrombosis. Catheter-directed thrombolysis combined with angioplasty was observed to be an effective and safe method for the treatment of hepatic vein obstruction in BCS complicated by thrombosis.

## Introduction

Budd-Chiari syndrome (BCS) is a series of disorders defined as obstruction of the hepatic veins and/or the proximal inferior vena cava (IVC). In western countries, BCS usually results from hypercoagulability (including myeloproliferative disorders, oral contraceptives and pregnancy)-induced thrombosis in one to three hepatic veins, or even in the IVC, which is characterized by an acute onset (1). It has been demonstrated that these patients may be treated with anticoagulation and thrombolytic (streptokinase) therapies to dissolve the thrombi in the hepatic veins (2,3). However, in eastern countries, membranous obstruction of the IVC and/or the hepatic veins is the most frequently occurring underlying cause of BCS (4), and this is most likely induced by organized thrombi or congenital factors. In eastern countries, BCS is a chronic condition and patients are treated with balloon dilatation or stent placement (5). In addition, a transjugular intrahepatic portosystemic shunt (TIPS) or alternative surgical treatment (including shunting procedures and liver transplantation) may be performed in patients with diffuse hepatic venous occlusion (6,7).

The incidence of BCS complicated by IVC thrombosis is 5% (8); however, BCS complicated by hepatic vein thrombosis, which classically manifests as acute or chronic episodes of ascites and liver failure, is relatively rare and has a poor prognosis. It has been demonstrated that, for simple hepatic vein membranous or segmental occlusion in BCS without thrombosis, balloon dilatation or stenting treatment may result in a satisfactory therapeutic efficacy (9,10). Anticoagulant and systemic thrombolytic therapy may be conducted for hepatic vein thrombosis in BCS without membranous or segmental occlusion (2,3). In contrast with the previously mentioned lesions, membranous or segmental occlusion of the hepatic vein and hepatic vein thrombosis co-exist in patients with BCS in which the hepatic vein obstruction is complicated by thrombosis. Therefore, there is no blood flow in the hepatic veins, due to hepatic vein obstruction and thrombosis, which presents a challenge with regard to the use of systemic thrombolysis to dissolve the clots in the hepatic veins. According to previous studies, a TIPS and liver transplantation are able to successfully treat BCS complicated by hepatic vein thrombosis (11,12). However, to date, there have been relatively few reports regarding the application of catheter-directed local thrombolysis combined with balloon dilatation or stent placement in the hepatic veins in the treatment of hepatic vein obstruction in BCS complicated by thrombosis. From February 2006 to April 2012, we treated 14 patients with BCS complicated by hepatic vein thrombosis by performing a catheter-directed local injection of urokinase into the hepatic veins, combined with balloon dilatation or stent placement. Certain patients underwent a predilatation of the occluded hepatic veins prior to the catheter-directed thrombolysis. The aim of the study was to assess the efficacy and safety of this method in the treatment of BSC-hepatic vein obstruction complicated by thrombosis.

## Materials and methods

### Clinical information

A total of 14 patients, including six males and eight females, with an average age of 34±10.3 years (range, 15–55 years), were recruited for this study. The course of the disease in the patients ranged between ten days and eight years. With regard to the clinical manifestations, there were 14 cases of abdominal distension and stubborn ascites; one case of upper gastrointestinal hemorrhage; five cases of varicose abdominal veins; two cases of leg swelling; one case of varicose veins in the lower limbs and one case of pigmentation and ulceration in the lower limbs. All the cases were diagnosed as BCS complicated by hepatic vein thrombosis, as indicated by color Doppler ultrasound, computed tomography angiography (CTA), magnetic resonance angiography (MRA) or digital subtraction angiography (DSA). Among the 14 cases, there were eight and six cases of acute and subacute thrombosis, respectively (Table I). One patient (case 1) had undergone an IVC stent placement seven years previously and another patient had received a mesoatrial shunt four years previously. This study was conducted in accordance with the Declaration of Helsinki and with approval from the Ethics Committee of the Affiliated Hospital of Xuzhou Medical College (Xuzhou, China). Written informed consent was obtained from all participants.

### IVC and hepatic vein angiography

Following the puncture of the femoral vein and/or the internal jugular vein, the IVC angiography was performed by inserting a 5F pigtail catheter to examine whether the IVC was clear and whether thrombosis was apparent. Subsequent to this, a 5F angiographic catheter (Tempo^®^-4; Cordis Corporation, Miami, FL, USA) was inserted into the hepatic vein or the accessory hepatic vein to replace the pigtail catheter, using the soft end of a smooth guide wire. If the insertion was not successful, then a steel needle with an arc-shaped head-end was utilized to puncture and open the occluded hepatic vein, followed by the insertion of the 5F angiographic catheter into the hepatic vein or the accessory hepatic vein to perform the angiography. Through the angiography, it was possible to determine the size of the thrombus, the collateral circulation status within the hepatic vein or the accessory hepatic vein and the occlusion status at the proximal end of the vein.

### Catheter-directed urokinase thrombolysis

In all the cases, a 5F pigtail or infusion catheter (cat. no. N5.0-35-100-P-16S-0-RIS-8.0/16.0; Cook Medical, Inc., Bloomington, IN, USA) was inserted into the hepatic vein via the jugular vein. A balloon with a diameter of 8 mm was used to predilate the occluded hepatic vein, prior to the thrombolysis by the indwelling catheter, in the six patients with subacute thrombus in the hepatic vein. In one case, a 5F infusion catheter was inserted into the accessory hepatic vein through the femoral vein. A total of 100,000 units urokinase in 20 ml saline was pulse-spray injected through the catheter once every 3–6 h. During the thrombolytic therapy, 5,000 units low-molecular-weight heparin sodium was injected subcutaneously every 12 h. Based on the angiography reviews performed every two days, the position of the catheter was adjusted according to the thrombolytic status to ensure that the sidehole region of the catheter was always in contact with the thrombus. The criteria for ending the therapy were: i) the thrombus was completely dissolved; ii) the thrombus was consistent in two consecutive reviews; and iii) internal bleeding was observed or other complications occurred.

### Balloon dilation and/or stent placement

Following the thrombolytic therapy, balloons with a diameter ranging between 10 and 14 mm were inserted into the hepatic or accessory hepatic veins of the patients. Five patients were fitted with a stent measuring 10–14 mm in diameter (Bard International, Inc., Murray Hill, NJ, USA) due to the balloon dilation resulting in an unsatisfactory reduction of the hepatic vein pressure or a retraction rate of the vein dilation of >50%. In one patient with a thrombus in the accessory hepatic vein, the balloon dilation and stent placement were performed under the protection of an Antheor™ temporary vena cava filter (Boston Scientific, Natick, MA, USA). A balloon with a diameter of 26 mm was used to dilate the narrow section of the IVC in two patients.

### Pressure monitoring

A piezometric tube was utilized to monitor the pressure of the hepatic vein, IVC and right atrium prior to and following the interventional therapy.

### Follow-up studies

The hepatic vein and IVC angiography were performed immediately subsequent to the operation. An ultrasound examination of the liver was conducted 1 week and 1, 3, 6 and 12 months following the operation, as well as each year subsequently, in order to evaluate the condition of the hepatic vein and IVC. The patients were prescribed oral warfarin for anticoagulation from the second day following operation for 24 months and the dose of warfarin was adjusted to maintain the international normalized ratio (INR) between two and three. In addition, a second angioplasty was performed in the patients with hepatic vein and IVC restenosis.

### Statistical analysis

The paired Student’s t-test was utilized to compare the pressure gradient between the hepatic vein and the right atrium prior to and following the interventional therapy. P<0.05 was considered to indicate a statistically significant difference.

## Results

### Treatment results

**Figure 1.**
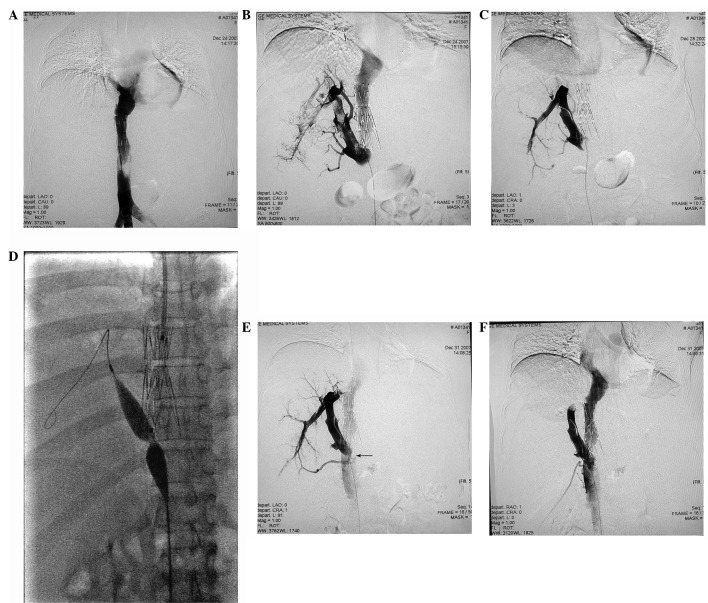
Case 1: Female, 37 years old, with Budd-Chiari syndrome combined with acute thrombosis in the accessory hepatic vein (AHV). Stent placement had been performed in the inferior vena cava (IVC) seven years previously by another hospital. (A) Stent region shown by IVC angiography: The blood stream was unobstructed within the stent, while stenosis occurred at the distal end of the vessel (indicated by the arrow). (B) In the AHV angiography performed through the femoral vein, a perforated membranous occlusion was shown at the opening of AHV, and a filling defect was generally observed in the vessel cavity. (C) Four days following the indwelling catheter thrombolytic therapy, the thrombus had predominantly dissolved, and only a small quantity of the embolus had shifted to the opening of AHV and blocked it (indicated by the arrow). (D) Four days following the thrombolytic therapy, a temporary filter was inserted through the jugular vein and a balloon catheter with a diameter of 10 mm was inserted through the femoral vein, by which the opening of the AHV was dilated. (E) Following the balloon dilation, the opening of the AHV remained narrow (indicated by the arrow). (F) Following the placement of a 12–40 mm stent, the vein cavity became unobstructed.

**Figure 2.**
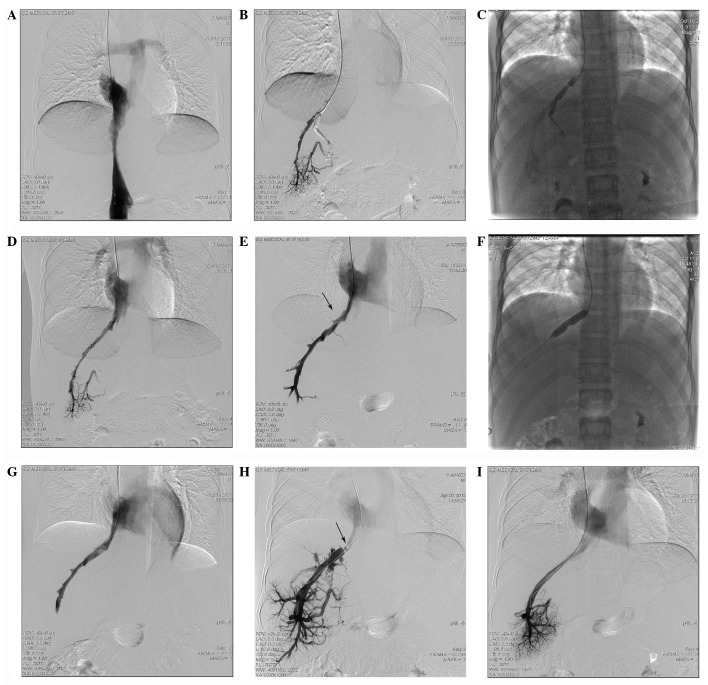
Case 13: Male, 15 years old, with Budd-Chiari syndrome in which right hepatic vein obstruction was combined with subacute thrombosis. (A) Inferior vena cava (IVC) angiography showed the blood stream to be unobstructed. (B) Right hepatic vein (RHV) angiography performed through the jugular vein showed that the proximal end of the RHV was blocked, and a filling defect was generally observed in the distal end of the cavity. (C) A balloon with a diameter of 8 mm was used to dilate the proximal end of the RHV. (D) An indwelling catheter was inserted into the RHV to perform the thrombolytic therapy. (E) Four days following the thrombolytic therapy, the thrombus in the RHV had predominantly dissolved, although the opening region remained narrow (indicated by the arrow). (F) Seven days following the thrombolytic therapy, a balloon with a diameter of 14 mm was used to dilate the proximal end of the RHV. (G) The thrombus in the RHV was completely dissolved and the blood stream became unobstructed. (H) Angiography was performed six months subsequently, and restenosis was observed at the opening region of the RHV (indicated by the arrow). (I) Blood flow became unobstructed in the RHV following the second balloon dilation.

There were 13 cases in which the treatment had a 100% success rate, with the time required for thrombolysis ranging from three to eight days (average, 5.2±1.7 days). The thrombi were completely and partially dissolved in nine and four cases, respectively; one hepatic vein was recanalized in 12 patients (right hepatic vein, seven cases; left hepatic vein, three cases; middle hepatic vein, one case and accessory hepatic vein, one case) (Fig. 1, case 1 and Fig. 2A–G, case 13), while two hepatic veins (right and left) were recanalized in one patient. The pressure gradient between the hepatic vein and the right atrium prior to interventional therapy was 18–41 cmH_2_O (average, 31.1±7.3 cmH_2_O; 1 cmH_2_O= 0.098kPa), which decreased to 3–11 cmH_2_O (average 6.8±2.7 cmH_2_O; t=15.1, P<0.01) following the procedure. There were no serious complications, such as bleeding or pulmonary embolism. The IVC was patent following balloon dilatation in two patients with IVC stenosis. Balloon dilatation was not performed in two patients with enlargement of the liver caudate lobe inducing IVC stenosis. The treatment was unsuccessful in one case, where a fatal liver tissue fracture-induced bleeding occurred during the catheter-directed thrombolysis treatment.

### Follow-up results

A total of 13 patients were followed up for 2–60 months (average, 24.8±19.6 months), among which one patient presented with hepatic vein restenosis six months subsequent to the interventional procedure. The patient was successfully treated with a second balloon dilatation (Fig. 2H and I, case 13). There was no recurrence of restenosis or thrombosis in the remaining 12 patients (Table II).

## Discussion

BCS complicated by thrombosis is relatively rare and the corresponding treatment includes systemic thrombolysis, agitation thrombolysis, TIPS and liver transplantation (10–15). In the present study, hepatic vein thrombosis was present in 14 patients with proximal hepatic vein obstruction. Catheter-directed thrombolysis was performed to remove the thrombi, followed by balloon dilatation and/or stent placement to recanalize the obstructed hepatic veins. The treatment was considered to have achieved technical and clinical success in 13 of the 14 patients. The hepatic veins were patent without serious complications, such as bleeding or pulmonary embolism, following the first and second interventional therapies.

The pathogenesis of BCS complicated by hepatic vein thrombosis has not yet been elucidated. Numerous factors, including malignancy, myeloproliferative disease, rheumatological disorders, hypercoagulability, infection and ulcerative colitis are potential etiological factors of thrombosis. However, the causes remain unidentified in 16–35% cases (1–3,16). Kuo *et al* (17) studied three cases of BCS complicated by hepatic vein thrombosis, in which the hepatic veins were recanalized without stenosis or occlusion following the removal of the thrombus by thrombolysis. In the present study, a residual membranous or segmental hepatic vein obstruction was apparent in 13 patients following the successful removal of the thrombus by thrombolysis, indicating that the hepatic vein thrombosis was secondary to hepatic vein obstruction. This finding differed from that in the study by Kuo *et al* (17). Following the obstruction of the hepatic veins, a number of factors, including slow blood flow, eddy formation and blood flow reversal, act in combination to promote thrombosis.

There are various treatments for BCS complicated by hepatic vein thrombosis. Dacha *et al* (1) described one case of BCS with hepatic vein thrombosis, the clots in the hepatic veins were partially dissolved with anticoagulation therapy. This case of BCS occurred due to thrombosis in the hepatic vein, without membranous or segmental occlusion; therefore the patient was different from the study. Thrombolytic therapy is important in the treatment of BCS with thrombosis. The drugs for thrombolysis consist of urokinase, streptokinase and recombinant tissue plasminogen activator (rt-PA). The methods utilized to deliver these drugs include systemic administration, injection through the hepatic artery and portal vein and transcatheter insertion into the hepatic vein (1,17,18). Sharma *et al* (18) performed thrombolytic therapy 12 times in 10 patients with acute BCS; of these 10 patients, three were treated with the thrombolytic drugs systemically, one was injected through the hepatic artery, four received the drugs by a local injection in the hepatic veins and two were locally treated with the drugs through the portal veins/TIPS. Among the patients who were administered with the drugs systemically or through the hepatic artery, the treatment was considered to be a partial success in only one patient; however, treatment was successful in five patients who received the injection locally through a TIPS or through the hepatic veins. In the present study, local thrombolysis was performed in the hepatic veins in 13 patients, and the clots were dissolved completely and partially in nine and four cases, respectively. Similar to the treatment of deep venous thrombosis in the lower limbs, the efficacy of thrombolysis was correlated with the time of the thrombosis (acute or subacute) and the application of a sidehole catheter for contiguous thrombolysis (19,20). There are certain advantages to utilizing a sidehole catheter for contiguous thrombolysis in hepatic veins: i) The presence of side holes on the catheter increases the contact area of the thrombolytic drug with the clots; and ii) the thrombolytic drugs are directly injected into the clots, which enhances the drug concentration and decreases the drug dose required, thereby reducing the risk of complications, such as bleeding.

Predilatation is important in the treatment of BCS complicated by IVC or hepatic vein thrombosis. Ding *et al* (21) performed predilatation (balloon diameter, 12–16 mm) in 13 cases of BCS with IVC obstruction complicated by thrombosis prior to the thrombolytic therapy: All the treatments were successful and no complications were observed. In the present study, predilatation was performed in the occluded hepatic veins of six patients using a balloon with a diameter of 8 mm, prior to the catheter-directed thrombolysis. These six patients were diagnosed with subacute thrombosis through color Doppler ultrasound. The purpose of predilatation is to partially recanalize the obstructed hepatic veins and restore the blood flow in the hepatic veins with thrombosis, which helps to improve the efficacy of the thrombolytic therapy. One potential complication of predilatation is pulmonary embolism, caused by the dislodging of a thrombus. There were no complications, such as pulmonary embolism, in the present study due to the following factors: i) Predilatation was only performed in patients with subacute thrombosis, in which the thrombi were unlikely to be dislodged due to their adhesion to the walls of the hepatic veins; ii) following the dilatation of the 8 mm-diameter balloon, an apparent lumen retraction (>50%) was observed at the proximal end of the hepatic veins, which made it more difficult for large clots to travel to the pulmonary arteries; iii) the following catheter-directed thrombolysis was able to gradually dissolve any clots in a wide radius around the side holes of the catheter in the hepatic veins.

In the current study, one patient presented with a perforated membrane occlusion of the accessory hepatic vein complicated by acute thrombosis. During the thrombolytic treatment, the emboli moved to the opening of the accessory hepatic vein, and therefore the balloon dilatation and stent placement in the accessory hepatic vein were performed under the protection of a temporary filter, which avoided pulmonary embolism.

There was one fatality during the catheter-directed thrombolytic treatment due to liver tissue fracture-induced bleeding. It was difficult to distinguish the liver parenchyma from extensive thrombosis in the hepatic vein during the detection of the hepatic vein by the catheter, and, as a result, the catheter went too deep and punctured the distal hepatic vein end through to the liver parenchyma. The catheter was subsequently retained in the hepatic vein for urokinase thrombolysis, which led to liver parenchyma fracture and bleeding.

Several methods, including TIPS (11), surgical shunt and liver transplantation (12) may be utilized to treat BCS complicated by hepatic vein thrombosis. In contrast with the previously mentioned studies, the present study successfully applied local thrombolysis, balloon dilatation and/or stent placement in 13 patients. This therapy restored the blood flow to the hepatic veins through thrombolysis and percutaneous transluminal angioplasty (PTA), which was consistent with the physiological status in the treatment of BCS.

There were a number of limitations to the present investigation, including the fact that it was a single center retrospective study with few patients. Furthermore, the study did not include a long-term follow-up. To provide more conclusive results, it may be necessary to increase the number of patients and perform randomized controlled studies, in addition to observing the long-term follow-up results of catheter-directed thrombolysis combined with angioplasty in the treatment of hepatic vein obstruction in BCS complicated by thrombosis.

In conclusion, catheter-directed thrombolysis was performed to remove thrombi, followed by balloon dilatation and/or stent placement to recanalize the hepatic veins, which corresponded well with the physiological status. Predilatation and multiple sidehole catheters were able to promote the efficacy of the thrombolysis. The preliminary results indicated that catheter-directed thrombolysis combined with angioplasty was an effective and safe method for the treatment of hepatic vein obstruction in BCS complicated by thrombosis and that the short- or mid-term efficacy of the treatment was reliable. However, the long-term efficacy of the treatment requires further observation.

## Figures and Tables

**Table I.** General patient data.

Case no.	Gender (M/F)	Age (years)	HV with proximal occlusion and combined thrombosis	HV with diffuse occlusion	Thrombosis property	IVC status
1	F	37	RHV, MHV, LHV, AHV	None	Acute	Stenosis
2	F	42	RHV, LHV	MHV	Acute	Clear
3	F	55	RHV, MHV, LHV	None	Acute	Clear
4	M	42	RHV, MHV, LHV	None	Subacute	Stenosis
5	F	28	RHV	MHV, LHV	Acute	Clear
6	F	42	RHV, MHV, LHV	None	Subacute	Clear
7	M	27	MHV, LHV	RHV	Acute	Clear
8	F	25	RHV, LHV	MHV	Subacute	Clear
9	M	34	RHV, MHV, LHV	None	Acute	Clear
10	M	28	RHV	MHV, LHV	Acute	Stenosis
11	F	31	RHV, MHV, LHV	None	Subacute	Clear
12	F	40	RHV, MHV, LHV	None	Acute	Clear
13	M	15	RHV	MHV, LHV	Subacute	Stenosis
14	M	23	RHV, MHV	LHV	Subacute	Clear

M, male; F, female; IVC, inferior vena cava; HV, hepatic vein; RHV, right HV; MHV, middle HV; LHV, left HV; AHV, accessory HV.

**Table II.** Patient status following treatment.

Case no.	Thrombolysis period (days)	Urokinase dosage (units)	HV intervention		Treated HV	Follow-up (months)	Outcome
			Balloon (mm)	Stent (mm)			
1	7	280×10^4^	10–40	12–40	AHV	60	No recurrence
2	7	210×10^4^	15–40	14–40	RHV	55	No recurrence
3	3	180×10^4^	10–40	-	LHV	48	No recurrence
4	5	200×10^4^	12–40	-	RHV, LHV	18	No recurrence
5	4	160×10^4^	14–40	12–40	RHV	38	No recurrence
6	8	320×10^4^	12–40	-	LHV	12	No recurrence
7	4	160×10^4^	12–40	-	RHV	9	No recurrence
8	6	260×10^4^	14–40	16–40	MHV	6	No recurrence
9	5	200×10^4^	12–40	-	LHV	25	No recurrence
10	3	120×10^4^	14–40	14–40	RHV	26	No recurrence
11	5	180×10^4^	16–40	-	RHV	17	No recurrence
12	3	150×10^4^	-	-	-	-	Fatality
13	7	280×10^4^	14–40	-	RHV	6	Restenosis, second PTA
14	6	240×10^4^	16–40	-	RHV	2	No recurrence

HV, hepatic vein; AHV, accessory HV; RHV, right HV; LHV, left HV; MHV, middle HV; PTA, percutaneous transluminal angioplasty.
